# Supplementary material for: Estimating intra- and inter-subject oxygen consumption in outdoor human gait using multiple neural network approaches
Source: PLoS One. 2024 Sep 27;19(9):e0303317. doi: 10.1371/journal.pone.0303317 (PMC11432871; doi:10.1371/journal.pone.0303317)
Supplement: S1 Table — Networks are ordered in ascending order with respect to their average RMSE. (PDF) [file pone.0303317.s001.pdf]

Supporting information for Müller et al. – "Estimating intra- and inter-subject oxygen consumption in outdoor human gait using multiple neural network approaches"

No.	Network type	RMSE (mean)	RMSE (std)	part. spec.	seq. len.	model configurations
1	XceptionNet	2.4295	0.2128	TRUE	200	n_f=16, c_{out} =16
2	XceptionNet	2.6109	0.3832	FALSE	200	n_f=16, c_{out} =16
3	XceptionNet	2.6262	0.3616	TRUE	200	n_f=16, c_{out} =32
4	XceptionNet	2.7328	0.5506	FALSE	200	n_f=16, c_{out} =32
5	XceptionNet	2.7533	0.4526	TRUE	200	n_f=8, c_{out} =32
6	XceptionNet	3.0478	0.8670	TRUE	200	n_f=8, c_{out} =16
7	XceptionNet	3.0600	0.6613	FALSE	200	n_f=8, c_{out} =32
8	ResNet	3.0751	0.5092	TRUE	200	n_f=24, c_{out} =16, kss= $[3, 3, 3]$
9	RNN	3.0865	0.1078	TRUE	200	one-directional GRU
10	ResNet	3.1489	0.4169	FALSE	200	n_f=24, c_{out} =16, kss= $[7, 5, 3]$
11	CNN 16	3.3202	0.5633	FALSE	200	
12	RNN	3.3328	0.7950	FALSE	200	one-directional GRU
13	ResNet	3.3496	0.7900	TRUE	200	n_f=24, c_{out} =16, kss= $[7, 5, 3]$
14	CNN 16	3.3694	0.3840	TRUE	200	
15	RNN	3.3844	0.4231	TRUE	200	bi-directional RNN
16	RNN	3.4680	0.6242	FALSE	200	bi-directional GRU
17	RNN	3.4716	0.4429	FALSE	200	bi-directional RNN
18	RNN	3.4911	0.4315	FALSE	200	one-directional LSTM
19	RNN	3.5010	0.7129	FALSE	200	bi-directional LSTM
20	XceptionNet	3.5048	0.1497	FALSE	50	n_f=16, c_{out} =16
21	ResNet	3.5128	0.5988	FALSE	200	n_f=24, c_{out} =16, kss= $[3, 3, 3]$
22	ResNet	3.5486	0.6032	TRUE	50	n_f=24, c_{out} =16, kss= $[3, 3, 3]$
23	RNN	3.5719	0.6276	TRUE	200	one-directional LSTM
24	CNN	3.6048	0.8707	FALSE	200	
25	RNN	3.6962	0.5952	TRUE	200	bi-directional LSTM
26	ResNet	3.7157	0.2107	FALSE	50	n_f=24, c_{out} =16, kss= $[3, 3, 3]$
27	RNN	3.7365	0.4748	FALSE	200	one-directional RNN
28	RNN	3.7460	0.7335	TRUE	200	bi-directional GRU
29	ResNet	3.8792	0.7209	FALSE	50	n_f=24, c_{out} =16, kss= $[7, 5, 3]$
30	ResNet	3.8856	0.8442	TRUE	50	n_f=24, c_{out} =16, kss= $[7, 5, 3]$
31	RNN	3.9168	0.5130	TRUE	200	one-directional RNN
32	DenseNet	3.9190	0.9900	FALSE	200	
33	XceptionNet	4.0829	0.7036	TRUE	50	n_f=16, c_{out} =32
34	XceptionNet	4.0942	0.5790	TRUE	50	n_f=8, c_{out} =16
35	XceptionNet	4.1529	0.6883	FALSE	50	n_f=8, c_{out} =32
36	XceptionNet	4.3845	1.5622	FALSE	200	n_f=8, c_{out} =16
37	CNN	4.4999	1.2157	TRUE	200	
38	XceptionNet	4.6494	0.9080	FALSE	50	n_f=8, c_{out} =16
39	XceptionNet	4.8867	0.6928	TRUE	50	n_f=16, c_{out} =16
40	DenseNet	4.9339	0.6294	TRUE	50	
41	XceptionNet	5.0884	1.3449	FALSE	50	n_f=16, c_{out} =32
42	CNN	5.1862	0.3495	TRUE	50	
43	RNN	5.2494	0.4595	FALSE	50	bi-directional GRU
44	RNN	5.2790	0.8909	FALSE	50	bi-directional RNN
45	RNN	5.3120	0.5529	FALSE	50	one-directional GRU
46	RNN	5.3302	0.8224	FALSE	50	one-directional LSTM
47	RNN	5.3399	0.7750	FALSE	50	one-directional RNN
48	RNN	5.4531	1.2189	TRUE	50	bi-directional RNN
49	RNN	5.4962	0.7417	FALSE	50	bi-directional LSTM
50	RNN	5.6207	1.0542	TRUE	50	bi-directional GRU
51	RNN	5.6606	1.3026	TRUE	50	one-directional GRU
52	XceptionNet	5.7136	2.3692	TRUE	50	n_f=8, c_{out} =32
53	RNN	5.7538	1.1726	TRUE	50	one-directional LSTM
54	CNN 16	5.7692	1.3642	FALSE	50	
55	RNN	6.1492	2.0787	TRUE	50	one-directional RNN
56	RNN	6.3716	2.0295	TRUE	50	bi-directional LSTM
57	CNN 16	6.5172	1.9349	TRUE	50	
58	DenseNet	7.9411	9.1471	TRUE	200	
59	CNN	8.1928	3.8141	FALSE	50	
60	DenseNet	12.0831	8.9866	FALSE	50	

The table contains results for all 60 tested neural network configurations for inter-subject estimation of oxygen consumption. Column *Network type* shows the type of network, column *part. spec.* is TRUE if participant-specific features were used in the network and FALSE otherwise. Columns *seq. len.* and *model configurations* yield the length of input sequences and information on configuration hyperparameters respectively. Columns *RMSE (mean)* and *RMSE (std)* contain the average root mean square errors and the corresponding standard deviations. Networks are ordered in ascending order with respect to their average RMSE.
